# From local adaptation to activism and global solidarity: framing a research and innovation agenda towards true health equity

**DOI:** 10.1186/s12939-016-0492-8

**Published:** 2017-02-21

**Authors:** Eric A. Friedman, Lawrence O. Gostin

**Affiliations:** O’Neill Institute for National and Global Health Law, Georgetown University Law Center, 600 New Jersey Avenue NW, Washington, DC, 20001 USA

## Abstract

The proposal for a global health treaty aimed at health equity, the Framework Convention on Global Health, raises the fundamental question of whether we can achieve true health equity, globally and domestically, and if not, how close we can come. Considerable knowledge currently exists about the measures required to, at the least, greatly improve health equity. Why, then, do immense inequities remain? Building on this basic question, we propose four areas that could help drive the health equity research and innovation agenda over the coming years.

First, recognizing that local contexts will often affect the success of policies aimed at health equity, local research will be critical to adapt strategies to particular settings. This part of the research agenda would be well-served by directly engaging intended beneficiaries for their insights, including through participatory action research, where the research contributes to action towards greater health equity.

Second, even with the need for more local knowledge, why is the copious knowledge on how to reduce inequities not more frequently acted upon? What are the best strategies to close policymakers' knowledge gaps and to generate the political will to apply existing knowledge about improving health equity, developing the policies and devoting the resources required? Linked to this is the need to continue to build our understanding of how to empower the activism that can reshape power dynamics.

Today’s unequal power dynamics contribute significantly to disparities in a third area of focus, the social determinants of health, which are the primary drivers of today’s health inequities. Continuing to improve our understanding of the pathways through which they operate can help in developing strategies to change these determinants and disrupt harmful pathways.

And fourth, we return to the motivating question of whether we can achieve health equity. For example, can all countries have universal health coverage that comprehensively meets all of people’s health needs? How to foster the national and global solidarity to achieve such equity? The answers to questions such as these can help point the way to measures, often well outside the narrow realm of technical solutions, to realize the right to health, and to achieve and sustain substantive health equality.

## Background

Momentum grows for the Framework Convention on Global Health (FCGH), the proposed treaty based on the right to health aimed at achieving equity [1, 2]. The focus on a legal instrument as a means towards health equity reflects the great deal we already know about how to improve health equity, both strategies within the health system and beyond, including the power of law. A global treaty could help speed application of this knowledge. Indeed, one central component of the FCGH could be national health equity strategies to catalyze action on health inequities, examining causes of inequities for each marginalized population and developing budgeted action plans to overcome them [3]. This proposal rests on the assumption that between the global pool of knowledge, national experiences, and formative input of disenfranchised populations themselves, countries have enough understanding of paths towards greater equity that, if these understandings were applied, would make a significant difference [4].

For example, universalizing standard public health interventions like clean water, adequate sanitation, and immunizations will help populations in poorer countries achieve the dramatic health gains seen in wealthier countries over the last century. Removing user fees and other financial obstacles to health coverage, along with affirmative measures such as interpretation services, empowering people through health literacy, and assurances of non-discrimination can help achieve universal health coverage and enable all people to benefit from the wonders of modern medicine. Other interventions would focus on specific health needs, such as maternity homes (enabling expectant mothers in rural areas to be near health facilities at the end of their pregnancies), sufficient numbers and distribution of skilled birth attendants, and free transportation to reduce maternal mortality even among the poorest women.

Despite this knowledge of actions that could lead to far greater health equity, dramatic inequities persist. Based on one set of measures, more than one in three deaths globally can be linked to health inequities [5]. Why is this knowledge insufficiently translated into practice? We believe at least four factors are at the heart of the answer, and could help orient the health equity research and innovation agenda. First, widely applicable truths might not hold in certain circumstances, leading to the importance of understanding local context. Second, knowledge of what works is too often simply not applied, leading to questions pertaining to failure of state action and inequitable distribution of power and resources. This inequitable distribution is reflected in disparities in the social determinants of health, leading to a third area of focus, better understanding how to improve health through addressing these determinants. And fourth is the foundational question of how close we can come to substantive health equality within political and economic constraints – and how we can overcome these constraints. These issues may not be new, but we believe require a more central focus in the health research agenda.

First, even seemingly well-designed, pro-equity policies might prove ineffective due to the local context. For example, when health services are available and basic barriers like cost and distance are removed, such as free distribution of insecticide-treated bednets, or free immunizations provided in people’s communities, what might prevent uptake? This requires local research to uncover the answer, whether bednets are being used for fishing [6], community members distrust health workers, or patients are forced to provide informal payments. Randomized control trials are becoming one powerful tool for understanding the effectiveness of health interventions in particular settings [7]. Other strategies, including operational research and pilot projects, can help adapt strategies for health equity to disparate settings.

How best to understand why health interventions are or not working? Ask the people intended to benefit from them. Qualitative research assumes particular import. Most valuable may be participatory action research, a cycle of analysis, reflection, and action where research subjects are engaged as active partners in the research, and that directly empowers people to create change in their communities [8].

Second, even with these uncertainties, states could implement policies and devote the resources that we know would make tremendous progress towards health equity, but too often fail to do so. To what extent is this because policymakers lack knowledge about effective policies – in which case, how to ensure that policymakers have this knowledge? Where do current roadblocks rest in past policy choices, such as health insurance schemes that offer more benefits to formal sector workers than informal workers and indigent populations – in which case, how to transform these systems, from overcoming entrenched interests to managing a transition phase? What measures, from changing incentives to changing expectations, could end often pervasive corruption in the health sector? And how to transform health systems and the political processes that shape them to give greater voice to those who suffer the most severe health inequities? Gaining a better understanding of how to enhance the effectiveness and reach of a broad spectrum of health accountability measures, including creating spaces at community, national, and global levels that enable meaningful participation and that can create new power dynamics, promises to be central to this component of the health equity research agenda.

Change in these areas will require political will, which in turn will require activism. Mark Heywood has described activism as a determinant of health [9]. The success of the Treatment Action Campaign in forcing a reluctant South African government to begin AIDS treatment is testimony to this truth [10]. To continue to build our understanding of how to deploy this vital determinant and resource, research on health activism of all sorts – from action in the streets to litigation in courts, from action by community members and civil society organizations to action by allies within the government and health workforce – may be among the most important areas of health equity research.

Health activism is needed to mobilize political will. Yet political will might be lacking because “the inequitable distribution of power, money, and resources” keep the marginalized marginalized, beyond the central concern of the politically powerful, and left behind within their own communities [4]. These inequities are central to how people are affected by the social determinants of good health. This brings us to our third research area: What are the specific pathways that the social determinants of health take, and how can we most effectively intervene at as many points as possible along these pathways?

The social determinants of health operate at multiple levels. Some operate directly, themselves causing ill health, such as the reduced control that lower-income people tend to have in their work life, contributing to greater stress and, in turn, cardiovascular diseases [11]. Others operate indirectly at the personal level, where people’s socioeconomic status may make those who are worse off less able to undertake healthy behaviors (e.g., a nutritious diet, because of cost or limited access to healthier foods) and more likely to take undertake unhealthy behaviors (e.g., smoking, to relieve stress and as a small, affordable pleasure). Some are linked directly to the health system, such as subconscious biases of health professionals leading them to treat patients of different races or genders differently, in ways not based in evidence. They operate at the community level, where poorer communities live in more polluted areas. And they operate at the political level, affecting the political will to address health equities, and contributing to policies that exacerbate health inequities. We will need to understand these pathways and the measures required to affect them.

Fourth, we must explore the fundamental question of what degree of health equity we can achieve, a question centrally about politics and morality. Within our work on the FCGH, we have adopted the perspective of health equity towards health equality, with the view that we need to focus on how to redress health inequities, with a particular concern for those worst off, but that fundamentally all people enjoy the same right to the highest attainable standard of health, with the substantive equality this implies. Is it possible to demand and achieve health equality across all countries and segments of the population? What would be the policy implications?

Consider here one central part of the health equity agenda, universal health coverage. The World Health Organization has defined universal health coverage as “all people hav[ing] access to the health services they need (prevention, promotion, treatment, rehabilitation and palliative care) without the risk of financial hardship when paying for them” [12]. Can this be made real for all people, where even expensive yet demonstrably effective treatments are universally available? Universal health coverage might then exclude only interventions of unproven effectiveness and those not demonstrated to be more effective than less costly options. What would this vision of universal health coverage cost countries and the global community, and how to bring down these costs? What level of funding should be expected from lower-income countries, and what level of financial solidarity from higher-income countries? And an age-old question that our increasing global interdependence makes ever more important, how to foster that solidarity among us, within and among countries, to enable the necessary funding?

This aspect of the health equity research agenda would address other questions. For example, how to mobilize the political will to equitably share scarce medical technologies during a global pandemic? How to harness the health, economic, and other benefits we all receive from healthier populations to create the political imagination that substantive health equality should be our goal?

## Conclusion

New questions on health equity will continue to arise as health challenges evolve, such as climate change and the growing burden of non-communicable diseases. How to minimize the burden of these health threats on the poor and marginalized among us will also be part of the research agenda of the next 15 years, and will intersect with the four research areas we have posed, cutting edge scientific inquiries combining with fundamental questions of political will and human solidarity. The answers to these inquiries may often be difficult to implement, but necessary. And the answers can help point the way to measures, like the FCGH, to truly realize the right to health, and ultimately to achieve and sustain substantive health equality.

## Abbreviations

FCGH: Framework Convention on Global Health
